# What Does Postponement of Non-urgent Orthopedic Surgeries During the COVID-19 Pandemic Mean for Patients With Gonarthrosis in Turkey? A Qualitative Study

**DOI:** 10.7759/cureus.32448

**Published:** 2022-12-12

**Authors:** Seçkin Özcan

**Affiliations:** 1 Orthopedics and Traumatology, Yalova State Hospital, Yalova, TUR

**Keywords:** qualitative study, elective, gonarthrosis, pandemic, covid-19

## Abstract

Objective

During the COVID-19 pandemic, the surgical treatment of orthopedic diseases for elective surgical planning was suspended. This study aimed to evaluate patients' experiences with gonarthrosis regarding the postponement of non-urgent orthopedic surgeries during the COVID-19 pandemic.

Materials and Methods

This phenomenological qualitative study was carried out with patients diagnosed with grade 3 or 4 gonarthrosis in the orthopedics and traumatology outpatient clinic of a hospital in western Turkey between February 2021 and March 2021. Interviews with patients were conducted face-to-face using the semi-structured interview guide focusing on patients' feelings, thoughts, and experiences. Each participant was interviewed once. Data collection was halted based on the saturation of data. Data from semi-structured interviews with patients were analyzed using Colaizzi's content analysis method.

Results

The analysis of the study data yielded three themes: "lack of knowledge", "desperation" and "lack of opportunity to cure the gonarthrosis disease". Four sub-themes were identified under these three categories. While "not knowing what to do" and "unconscious drug use" were the sub-themes of the theme of lack of knowledge, "hopelessness" and "economic difficulties" were the sub-themes of the theme of desperation.

Conclusion

It was determined that patients were helpless due to difficulties accessing health services for other diseases during the COVID-19 pandemic. They experienced severe fear because it was unclear how long this process would continue.

## Introduction

COVID-19, a highly contagious infection, was first detected in China in December 2019 and spread worldwide within a few months. In Turkey, the first COVID-19 case was detected in March 2020 [1]. Because the virus spread rapidly around the world, there was a great crisis in all countries' health systems and the global economy. During this period, different data related to the pandemic process and its management were published worldwide [2]. In a crisis, many restrictions, which also have psychosocial effects implemented by many countries, including Turkish, to control the pandemic and ensure the effective functioning of the health system [3,4]. To use hospital bed capacities effectively, to reduce the risk of infection and to use the workforce of health professionals appropriately, rearrangements were made according to the COVID-19 emergency planning, and daily medical practice protocols were prepared. According to these protocols, the surgical treatment of orthopedic diseases for elective surgical planning was suspended [5].

All elective surgeries were postponed between March 2020 and June 2020 in Turkey, and the health system was adapted to the pandemic conditions. Along with the pandemic restrictions, elective surgeries were postponed in Turkey, and the physical therapy and rehabilitation units were closed. Therefore, only medical treatment options were used for the treatment of patients. After June 2020, the restrictions were gradually loosened, and surgeries were resumed in a controlled manner, according to the hospital conditions. However, the number of patients gradually increased with the addition of new patients to the waitlist during the pandemic period. The number of patients waiting for elective surgeries in the United Kingdom was 4 million before the pandemic, which increased to about 10 million [6]. Within the scope of COVID-19 precautions, arthroplasty surgeries were classified among the surgeries requiring urgency at the lowest level, and delaying these surgeries longer than three months was considered acceptable [7]. However, it is known that arthroplasty operations, which are performed more frequently due to the aging of the population worldwide, are one of the most commonly performed elective surgeries associated with orthopedics and traumatology [8]. According to the literature, the number of patients waiting for arthroplasty in the USA in July 2020 was approximately 500,000 [9].

It is known that the number of patients with osteoarthritis is more than 250 million people worldwide [10]. Gonarthrosis, a degenerative knee joint disease, is one of the most common types of osteoarthritis. Although medical treatment and physical therapy-rehabilitation applications are the priority options for these patients, surgical treatment is planned for patients with advanced-stage gonarthrosis, which causes them to suffer severe pain and lose some functions [11]. Although the cost of surgical treatment methods is high, it significantly improves patients' functional status, pain levels, and quality of life [12,13]. Elective surgeries are non-urgent surgeries. However, these surgeries should not be postponed for a long time because postponement of these surgeries leads to a decrease in the quality of life of the patients and causes them to suffer severe pain [14]. It is essential to understand what these patients, whose surgeries were postponed due to pandemic restrictions, were going through and how they felt. Therefore, this qualitative study aimed to explore the feelings and opinions of patients with gonarthrosis on treatment restrictions during the COVID-19 pandemic.

## Materials and methods

Study design

This Husserlian phenomenological qualitative study was conducted with patients diagnosed with grade 3 or 4 gonarthrosis. The main event in phenomenology is how the subject experiences events and the phenomenological method is done to reveal the experiences of the subjects included in the study.

Setting and participants

The study sample included patients diagnosed with gonarthrosis and accepted surgical treatment in a hospital's orthopedics and traumatology outpatient clinic in western Turkey between February 2021 and March 2021. In Turkey, elective surgeries were gradually allowed in June 2020 by taking the necessary precautions against COVID-19. However, this process was extended until March 2021 in the hospital where the study was conducted because the conditions were unsuitable. After March 2021, as the number of COVID-19 patients in the hospital began to decrease, elective surgeries were gradually resumed.

The participants were selected from patients diagnosed with gonarthrosis through purposive sampling. Patients with a diagnosis of grade 3 or 4 gonarthrosis and no previous history of surgery were included in this study. Seventy-two patients with grade 3 or 4 gonarthrosis were examined in the outpatient clinic during this period. All patients were invited to the outpatient clinic after giving information about the study by randomly calling by phone. In order not to cause any bias among the participants, they were called by the secretary working in the orthopedics and traumatology outpatient clinic. There were no patients who refused to participate in the study. Each participant was interviewed once. Data collection was continued until the obtained data reached saturation. In the 15th patient, it was decided that the data reached saturation (i.e., no new information was obtained, repeated information was obtained, and no further coding was available), and the interviews were terminated.

Data collection

Interviews with patients were conducted through the face-to-face interviewing method using the semi-structured interview guide focusing on the feelings, thoughts, and experiences of patients diagnosed with grade 3 or 4 gonarthrosis. The researcher created an interview guide based on the researcher's professional expertise and literature knowledge. The researcher, an orthopedic surgeon, treated patients with grade 3 or 4 gonarthrosis for eight years. The interview guide consisted of five main open-ended questions to assess patients' views on gonarthrosis and the impact of COVID-19 on the treatment process. The Personal Information Form included items questioning the demographic information of patients. Research questions are below: (1) What are your feelings and thoughts about your illness? (2) How do you think your condition has reached the current level? (3) How has the closure of physical therapy units due to COVID-19 affected you? Could you explain it? (4) How has the indefinite suspension of surgeries due to COVID-19 affected your treatment? Could you explain it? (5) What do you think about treating your disease at this stage?

To reduce the participants' concerns during in-depth interviews, the researcher asked them to express their feelings and listened to them quietly while talking. The researcher also communicated with the participants sensitively, respecting periods of silence and focusing on experiences and their readiness to continue with the interviews. The personal in-depth interviews lasted an average of 40 minutes (between 30 and 50 minutes). The interviews were audio recorded by the researcher, and the recordings were stored on his computer.

Data analysis

The researcher transcribed the voice recordings obtained during the interviews. Colaizzi's content analysis method was used in the data assessment to extract significant statements. The contents of the interviews were recorded with the Microsoft Office Word program, and the data obtained were analyzed in seven steps: (1) Transcribing all the participants' descriptions, reading and re-reading the transcript, (2) Extracting significant statements, (3) Creating formulated meanings, (4) Aggregating formulated meanings into theme Clusters, (5) Developing a detailed description, (6) Identifying the fundamental structure of the phenomenon, (7) Validation of the findings of the study through participant feedback [15].

The rigor of the study

To ensure the reliability of the study, the methods and the analyses used were described in detail. The data analysis after the interviews, what the participants said about the subject, and their facial expressions while they talked were considered. Apart from the researcher, coding was performed by two independent experts, which matched the themes created by the researcher. It is necessary to calculate intercoder reliability in encodings performed by more than one encoder. Therefore, Cohen's Kappa coefficient was calculated to evaluate intercoder agreement [16]. The fit ratio was 0.90 in the present study, indicating that the agreement between the researcher and independent experts was excellent. Since there was no personal relationship between the researcher and the participants, personal relationships did not affect the data collection processes.

Ethical considerations

Ethical approval to perform the study was obtained from Yalova University (Approval number: 2021/16, date of approval: January 05, 2021). The purpose of the research was explained to the participants, and written consent was obtained with forms prepared according to the Declaration of Helsinki. The participants were ensured that their names would be kept confidential. The names of the participants were coded as P1-P15.

## Results

The mean age of the participating patients was 69 (Min: 55; Max: 78) years. The majority of the participants were women and single. The sociodemographic data of the participants are shown in Table 1. It was determined that all the participants were retired, and their average income levels were below the poverty line of $1204.79 [17]. The feelings and thoughts of patients with gonarthrosis about the postponement of non-urgent surgeries during the COVID-19 pandemic were investigated.

**Table 1:** Data on the sociodemographic characteristics of the participants *Primary School Graduate

Participant	Age	Sex	Marital status	Educational status	Profession	Knowledge of the disease	Compliance with the treatment	Willingness to undergo surgery
P1	58	F	Widow	PSG^*^	Housewife	Yes	Yes	Yes
P2	68	F	Married	Illiterate	Housewife	Yes	Yes	Yes
P3	69	F	Widow	Illiterate	Housewife	Yes	Yes	Yes
P4	55	F	Widow	Illiterate	Housewife	Yes	Yes	Yes
P5	72	F	Widow	PSG	Housewife	Yes	Yes	Yes
P6	74	F	Widow	PSG	Housewife	No	No	Yes
P7	73	F	Widow	PSG	Housewife	Yes	Yes	Yes
P8	78	F	Widow	PSG	Housewife	Yes	No	Yes
P9	74	F	Widow	Illiterate	Housewife	Yes	Yes	Yes
P10	65	M	Married	PSG	Carpenter	Yes	Yes	Yes
P11	75	F	Widow	Illiterate	Housewife	Yes	Yes	Yes
P12	57	F	Widow	PSG	Housewife	Yes	Yes	Yes
P13	70	M	Married	PSG	Construction Worker	Yes	Yes	Yes
P14	75	F	Widow	PSG	Housewife	Yes	Yes	Yes
P15	71	F	Widow	Illiterate	Housewife	Yes	Yes	Yes

The results are presented in Table 2 as three themes and four sub-themes. The analysis of the study data yielded three themes: "lack of knowledge", "desperation" and "lack of opportunity to cure the gonarthrosis disease". Four sub-themes were identified under these three categories. While "not knowing what to do" and "unconscious drug use" were the sub-themes of the theme of lack of knowledge, "Economic difficulties" and "Hopelessness" were the sub-themes of the theme of desperation (Table 2).

**Table 2:** The themes and sub-themes produced are based on patients' experiences with gonarthrosis regarding the postponement of non-urgent orthopedic surgeries during the COVID-19 pandemic.

Themes	Sub-themes
1. Lack of knowledge	1.1 Not knowing what to do
	1.2 Unconscious drug use
2. Desperation	2.1 Hopelessness
	2.2 Economic difficulties
3. Lack of opportunity to cure the gonarthrosis disease	

Theme 1: Lack of knowledge

In the "lack of knowledge" theme, the patients' thoughts about their diseases and treatment processes were investigated. Under this theme, it was determined that the patients did not comply with the treatment because they did not know their illness, what caused it, and how it would be treated.

Sub-Theme 1.1: Not Knowing what to do

It was observed that the patients never thought about their disease, did not seek information about it, and all they wanted was to get rid of the pain as soon as possible. Some of the patients' statements were as follows: 

'...I know nothing about my disease, I have never thought about what causes it, what I should pay attention to... (P7)''.

 "…I do not understand what the doctor says, sometimes the medicines given by the doctor do not cure me at all, my pain does not go away, I stop taking the drugs… (P14)".

Sub-Theme 1.2: Unconscious Drug Use

It was defined as "unconscious drug use" as the patient's continuous use of drugs without the full knowledge of the effects or side effects, without the advice of a doctor. It was observed that the patients tended to take drugs unconsciously because surgeries were postponed due to the pandemic or they could not utilize physical therapy units since they were closed. Under the "unconscious drug use" theme, it was observed that the patients used drugs continuously without the doctor's advice to get rid of the severe pain they felt.

The majority of the patients expressed this situation as follows:

"…I didn't know what to do when I was in a lot of pain. I had a hard time coming to the hospital during the COVID-19 pandemic. To be frank, I was afraid of being infected with the coronavirus. I also used painkillers that I know myself. Afterwards, I noticed that my overuse of drugs led to side effects… (P1)".

"… I had to try many ways to get rid of my pain. I tried every drug when people around me said 'this helps'… (P2)".

Theme 2: Desperation

In this theme, it was determined that the patients experienced deep desperation and did not know what to do. The greatest cause of their desperation was that the path to surgery, which the patients considered the best solution for their disease, was closed.

The sub-themes created under this theme were "Economic difficulties" and "Hopelessness".

Sub-Theme 2.1: Economic Difficulties

Since elective surgeries continued in private hospitals during the pandemic, patients with better economic opportunities were presented to these hospitals. However, the patients who could not attain this opportunity continued to find solutions or take the drugs they knew. Some of the patients' statements were as follows:

 "… Someone I know told me that surgeries continue in private hospitals. How can I go there and have surgery? My means are not sufficient…" (P8).

"…If I had the opportunity, I would go to a private hospital and have my surgery, but I do not have such an opportunity. How can I get the necessary treatment in this case? … (P11).

Sub-Theme 2.2: Hopelessness

It was determined that the patients had concerns about the future and that the pain they suffered caused hopelessness. Most patients believed they could neither get rid of their pain nor undergo effective treatment. They stated that they could cope with their diseases more effectively, or at least they had the opportunity to have surgery before the COVID-19 pandemic. However, they seemed to have lost their hopes if the drugs did not help.

Some of the patients' statements were as follows:

"… The doctor said there is no cure for your problem. What can I do? I cannot have surgery. I don't know how much longer I will manage, how much longer I will endure this pain….(P5)".

"…Is there any other choice but to wait for this pandemic to be over? I will continue to be with the pain, because of my pain, you can't tell if it's night or day …(P10)

Theme 3: Lack of opportunity to cure the gonarthrosis disease

When the patients were asked about the cause of the disease and the best solution, it was determined that they did not pay any attention to their health, did not visit the doctor when necessary, but visited the doctor as a last resort when the pain became unbearable. While some of the patients wanted to have surgery, others were afraid of having surgery. Some of the patients' statements were as follows:

 "… I have no idea how this disease occurred, I don't know how it would go away. I don't like (avoid) visiting doctors, that's why I have never visited a doctor until today…" (P12). "… I don't know what this disease is or what its cause is, but all I want is the doctor's not asking me to have surgery. What I want is that he prescribes me medicine. I want to be well…" (P10). "…I do what the doctor asks me to do; if he asks me to have surgery, I undergo surgery, if he asks me to have physical therapy, I have physical therapy, but this pandemic has ruined me. I can neither have surgery nor benefit from medication. I can't get physical therapy either. I don't know how to get rid of this disease under these conditions...' (P11).

## Discussion

The COVID-19 pandemic has caused dramatic changes in orthopedic surgery practices in healthcare institutions worldwide [18]. During the pandemic, elective surgical operations were postponed indefinitely or planned months later, hoping the disease would disappear [19]. However, these surgeries are essential to relieve patients' pain, improve their quality of life, and prevent disease progression [14]. In the present study, the feelings and thoughts of patients with non-urgent gonarthrosis about the postponement of their surgeries during the COVID-19 pandemic were investigated.

Most of the participants in this study were postmenopausal women. According to the literature, gonarthrosis is more common in older women than older men [20]. The main reasons why gonarthrosis is more common in women are obesity, insufficient muscle strength, and decreased bone quality due to hormonal changes in the postmenopausal period [21,22]. In a study of women diagnosed with gonarthrosis, the stage of gonarthrosis was more advanced than it was in men, and the women experienced more pain than the men [23,24].

The participants were not knowledgeable enough about their diseases. They did not know what they should pay attention to during the treatment process, and they did not comply with the use of drugs. The patients were not knowledgeable enough about their diseases. They did not know what to pay attention to during the treatment process. During the COVID-19 pandemic, they used various drugs to relieve knee pain. The following statement made by the participants is the leading indicator that they tended to use inappropriate drugs "I tried every drug that people in my environment advised by saying 'this is good" to get rid of my pain ...". It is known that excessive use of drugs can bring about worse results and that one of the most common reasons patients seek medical care is the chronic pain they suffer [25]. In the USA, it is estimated that approximately 100 million patients are affected by chronic pain. Of these patients, 51% take inappropriate drugs to treat themselves because of the helplessness they experience [26]. In the literature, an increase in the use of opioids in patients due to postponed surgeries during the COVID-19 pandemic has been reported [6]. In a cross-sectional study conducted with 34186 patients on the preoperative use of opioids, it was reported that 23.1% of the patients who were scheduled for knee surgery took opioids preoperatively. Especially in patients waiting for hip surgery and knee arthroplasty, this rate varies between 40% and 44%. The prolongation of the surgical waiting period causes an increase in the number of opioids taken daily [27,28].

In the current study, the fact that physical therapy, rehabilitation, or surgical treatment was not provided for the patients who needed these services in a state hospital was the primary factor leading to the sufferance of these patients. In this process, it was observed that patients with insufficient economic means had difficulty accessing the ongoing elective health services in private hospitals and therefore experienced desperation. Most patients defined the current situation as follows: "…If I had the opportunity, I would go to a private hospital and have the surgery, but I do not have such an opportunity; in this case, how can I get the necessary treatment? …" It has been found that the cost of receiving health services from a private hospital was an indicator of prestige and was suitable only for patients with high socioeconomic status [29]. On the other hand, it is well known that the quality of life of patients who do not have the means and who wait to receive treatment decreases as the waiting period increases [30].

The COVID-19 pandemic poses a danger and despair, as many medically necessary surgeries have been delayed to preserve health resources and prevent disease transmission [19,24]. The statement "How can I get rid of this disease under these conditions" made by a patient who did not know when the COVID-19 pandemic would end and when we would switch to a normal surgery period was the most concrete indicator of the despair and hopelessness she suffered. In a study before the pandemic, patients defined the waiting period for total joint replacement surgery as 'worse than dying' [30]. According to a recent study, the number of patients describing the process as "worse than dying" while waiting for knee arthroplasty surgery nearly doubled during the COVID-19 outbreak. In the same study, 76.2% of the patients stated that their quality of life worsened while they waited for surgery [24].

Limitations of the study

The majority of the participants in the study were women, and their income levels were below the poverty line, which is a limitation of this study. The results obtained from the present study apply only to those who were surveyed.

## Conclusions

The present study revealed that patients who defined their knee-related problem as a severe disease were not knowledgeable enough about it. It was determined that knee pain significantly reduced their quality of life and caused them to suffer hopelessness and helplessness. It was also observed that they were helpless due to difficulties accessing health services during the COVID-19 pandemic. They experienced severe fear because it was unclear how long this process would continue. Because the present study is the first one conducted on this issue, it may guide professionals working in this field on how to approach patients, empathize with them and provide care for them.

Due to the effects of the indefinite postponement of elective surgeries during the COVID-19 pandemic on patients' quality of life, patients waiting to undergo surgery should be evaluated well. That patient-specific treatment plans should be made. In addition, understanding the feelings of patients on the surgery waitlist is very important because this will make the surgery bring about better outcomes and help the patient recover quickly. It can also affect the healing process of the patient positively. 
